# Supplementary material for: The capacity of origins to load MCM establishes replication timing patterns
Source: PLoS Genet. 2021 Mar 25;17(3):e1009467. doi: 10.1371/journal.pgen.1009467 (PMC8023499; doi:10.1371/journal.pgen.1009467)
Supplement: S1 Fig — yFS1059 cultures (replicates 1 and 2) were treated and synchronized as shown in Fig 1A before being released into S phase for replication timing measurements. yFS1075, yFS1020, and yFS1021 cultures were treated and synchronized as indicated in Fig 5A before being released into S phase for replication timing measurements. For yFS1075, flow cytometry data is shown for experiments where galactose-induced overexpression was performed for two hours or three hours. For yFS1020 and yFS1021, overexpression was performed for two hours. (PDF) [file pgen.1009467.s001.pdf]

Supplemental Figure 1

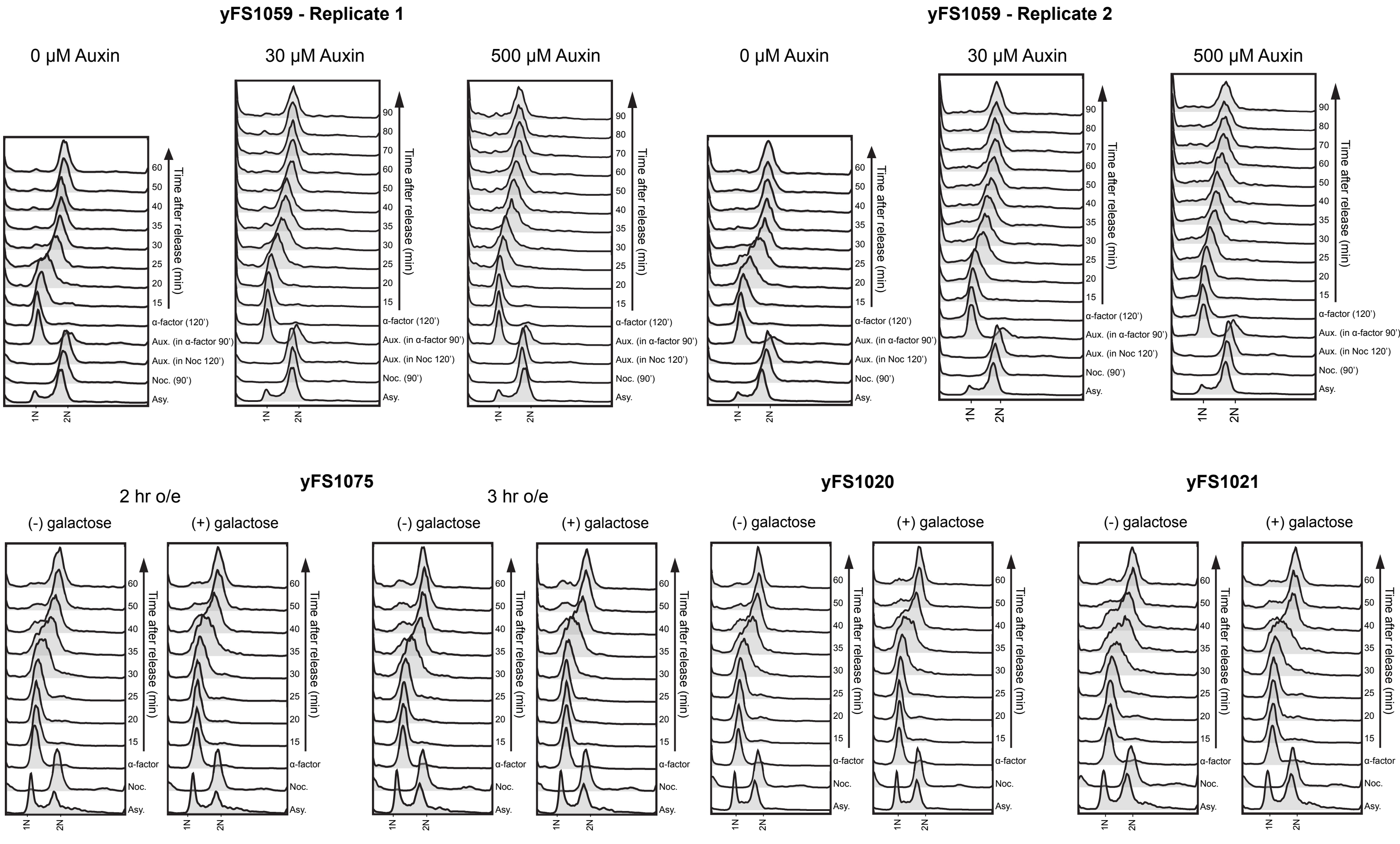

Supplemental Figure 1: Progression of synchronized cultures through S phase as monitored by flow cytometry

yFS1059 cultures (replicates 1 and 2) were treated and synchronized as shown in **Figure 1a** before being released into S phase for replication timing measurements. yFS1075, yFS1020, and yFS1021 cultures were treated and synchronized as indicated in **Figure 5a** before being released into S phase for replication timing measurements. For yFS1075, flow cytometry data is shown for experiments where galactose-induced overexpression was performed for two hours or three hours. For yFS1020 and yFS1021, overexpression was performed for two hours.
