# Supplementary material for: The capacity of origins to load MCM establishes replication timing patterns
Source: PLoS Genet. 2021 Mar 25;17(3):e1009467. doi: 10.1371/journal.pgen.1009467 (PMC8023499; doi:10.1371/journal.pgen.1009467)
Supplement: S2 Fig — a) For chronic down-regulation of MCM, exponentially-growing cultures of the indicated genotypes were serially diluted and spotted on plates containing 0 μM, 30 μM, or 500 μM auxin, with or without 100 mM hydroxyurea. OsTIR (yFS1044) refers to the background of the strain required for auxin-mediated degradation. WT = yFS833; MCM4-IAA17 = yFS1062; MCM4-IAA17-GFP = yFS1059; MCM4-GFP = yFS1082. b) For acute down-regulation of MCM, yFS1059 cultures were arrested at G1/S using α-factor as outlined in Fig 1A, washed, then serially diluted on YPD plates with or without 100 mM HU. (PDF) [file pgen.1009467.s002.pdf]

Supplemental Figure 2

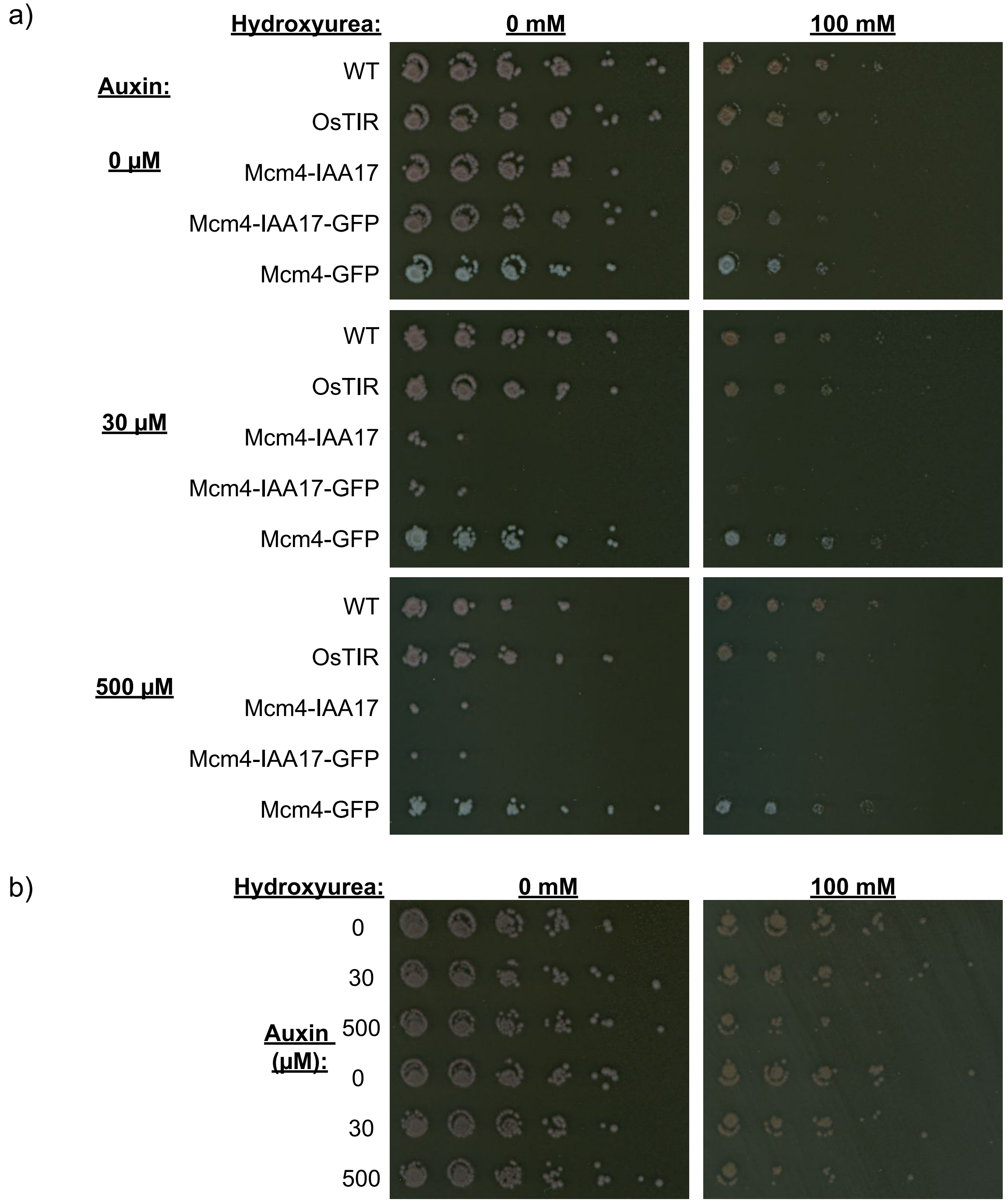

Supplemental Figure 2: Auxin-induced degradation of Mcm4 causes reduced viability

a) For chronic down-regulation of MCM, exponentially-growing cultures of the indicated genotypes were serially diluted and spoÇed on plates containing 0 μM, 30 μM, or 500 μM auxin, with or without 100 mM hydroxyurea. OsTIR (yFS1044) refers to the background of the strain required for auxin-mediated degradation. WT = yFS833; MCM4-IAA17 = yFS1062; MCM4-IAA17-GFP = yFS1059; MCM4-GFP = yFS1082.
b) For acute down-regulation of MCM, yFS1059 cultures were arrested at G1/S using α-factor as outlined in **Figure 1a**, washed, then serially diluted on YPD plates with or without 100 mM HU.
