# Supplementary material for: The capacity of origins to load MCM establishes replication timing patterns
Source: PLoS Genet. 2021 Mar 25;17(3):e1009467. doi: 10.1371/journal.pgen.1009467 (PMC8023499; doi:10.1371/journal.pgen.1009467)
Supplement: S3 Fig — a) MCM ChIP-seq signal at ARS origins in two biological replicates of yFS1059 for 0 μM, 30 μM, and 500 μM auxin treatments. b) Comparison of MCM signal at origins between the 0 μM auxin condition in this study and other publications [18,42]. c) Genome-wide replication timing correlation in 1 kb windows between the 0 μM auxin condition in this study and [59]. (PDF) [file pgen.1009467.s003.pdf]

Supplemental Figure 3

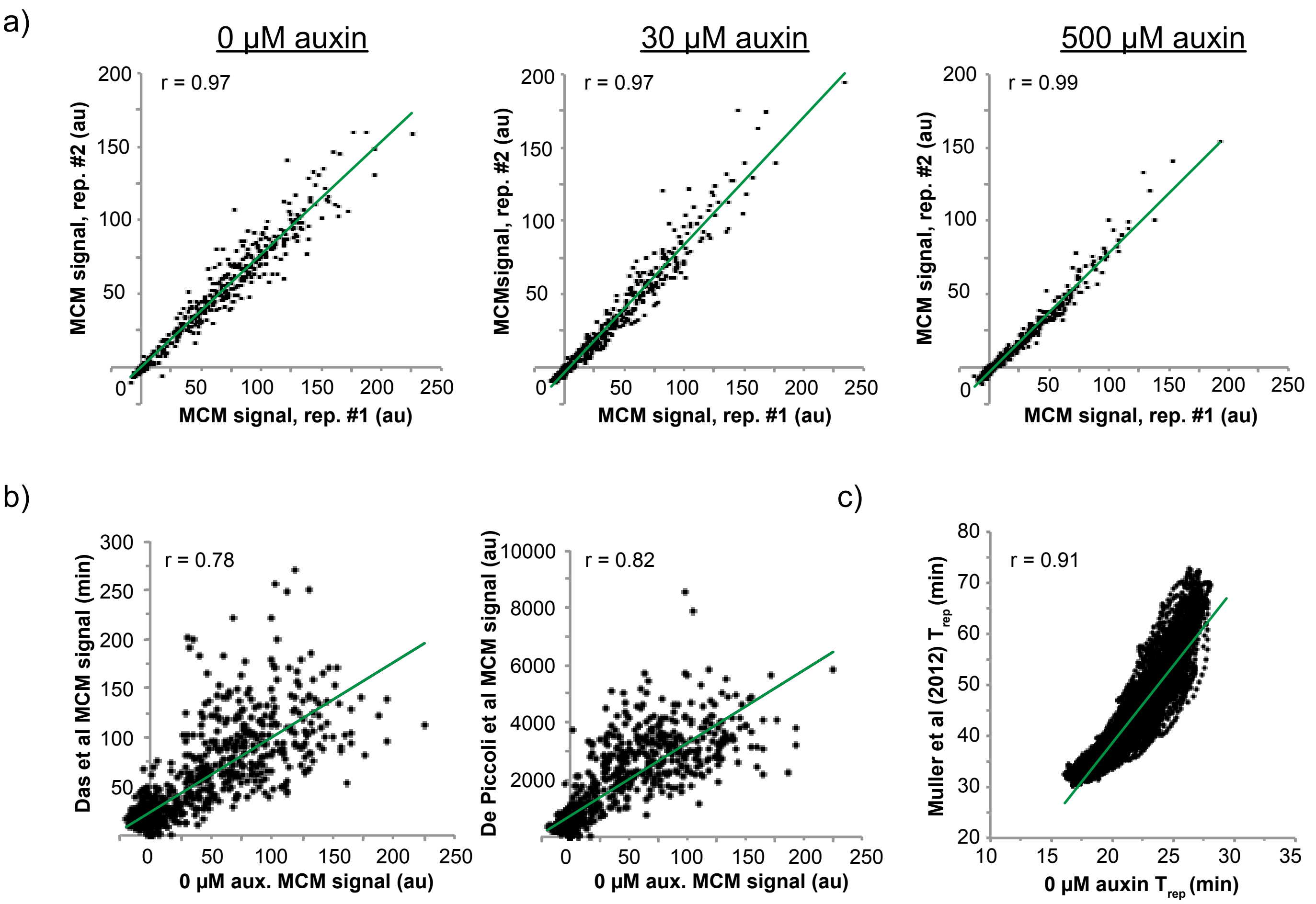

Supplemental Figure 3: MNase-ChIP-seq and replication timing results from the MCM reduction experiments are reproducible

a) MCM ChIP-seq signal at ARS origins in two biological replicates for 0 μM, 30 μM, and 500 μM auxin treatments (yFS1059 strain).
b) Comparison of MCM signal at origins between the 0 μM auxin condition in this study and other publications (Das et al., 2016, DePicoli et al., 2012).
c) Genome-wide replication timing correlation in 1 kb windows between the 0 μM auxin condition in this study and Mueller et al, 2012.
