# Supplementary material for: The capacity of origins to load MCM establishes replication timing patterns
Source: PLoS Genet. 2021 Mar 25;17(3):e1009467. doi: 10.1371/journal.pgen.1009467 (PMC8023499; doi:10.1371/journal.pgen.1009467)
Supplement: S4 Fig — a) The smallest number of origins required to make up 50% of the total MCM signal was calculated for each condition. The inter-origin distances for these sets of origins were calculated and plotted as a fraction of the total number of inter-origin distances for each condition. (PDF) [file pgen.1009467.s004.pdf]

Supplemental Figure 4

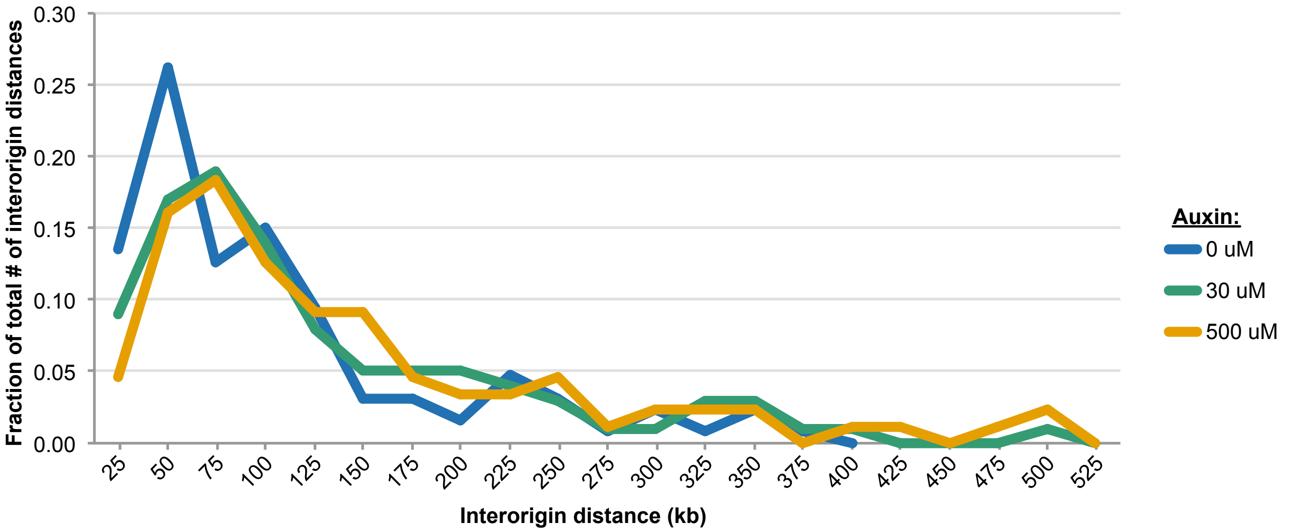

Supplemental Figure 4: Genome-wide reduction in MCM loading leads to larger inter-origin distances

The smallest number of origins required to make up 50% of the total MCM signal was calculated for each condition. The inter-origin distances for these sets of origins were calculated and plotted as a fraction of the total number of inter-origin distances for each condition.
