# Supplementary material for: The capacity of origins to load MCM establishes replication timing patterns
Source: PLoS Genet. 2021 Mar 25;17(3):e1009467. doi: 10.1371/journal.pgen.1009467 (PMC8023499; doi:10.1371/journal.pgen.1009467)
Supplement: S5 Fig — a) MCM signal at well-known and example single origins in response to auxin treatment (yFS1059—Replicate 2). Signal at the well-known ARS1 and ARS501 origins of replication shows slight reductions in response to treatment with 0 μM, 30 μM, and 500 μM auxin. ARS512 loses most of its MCM signal in response to auxin treatment, while ARS516 signal remains largely the same. b) Normalized MCM MNase-ChIP-seq density pro les centered at ACSs for the indicated auxin treatments (yFS1059—Replicate 2) for all, MCM-sized (50–90 bp) or nucelosome-sized fragments (125–165 bp). (PDF) [file pgen.1009467.s005.pdf]

Supplemental Figure 5

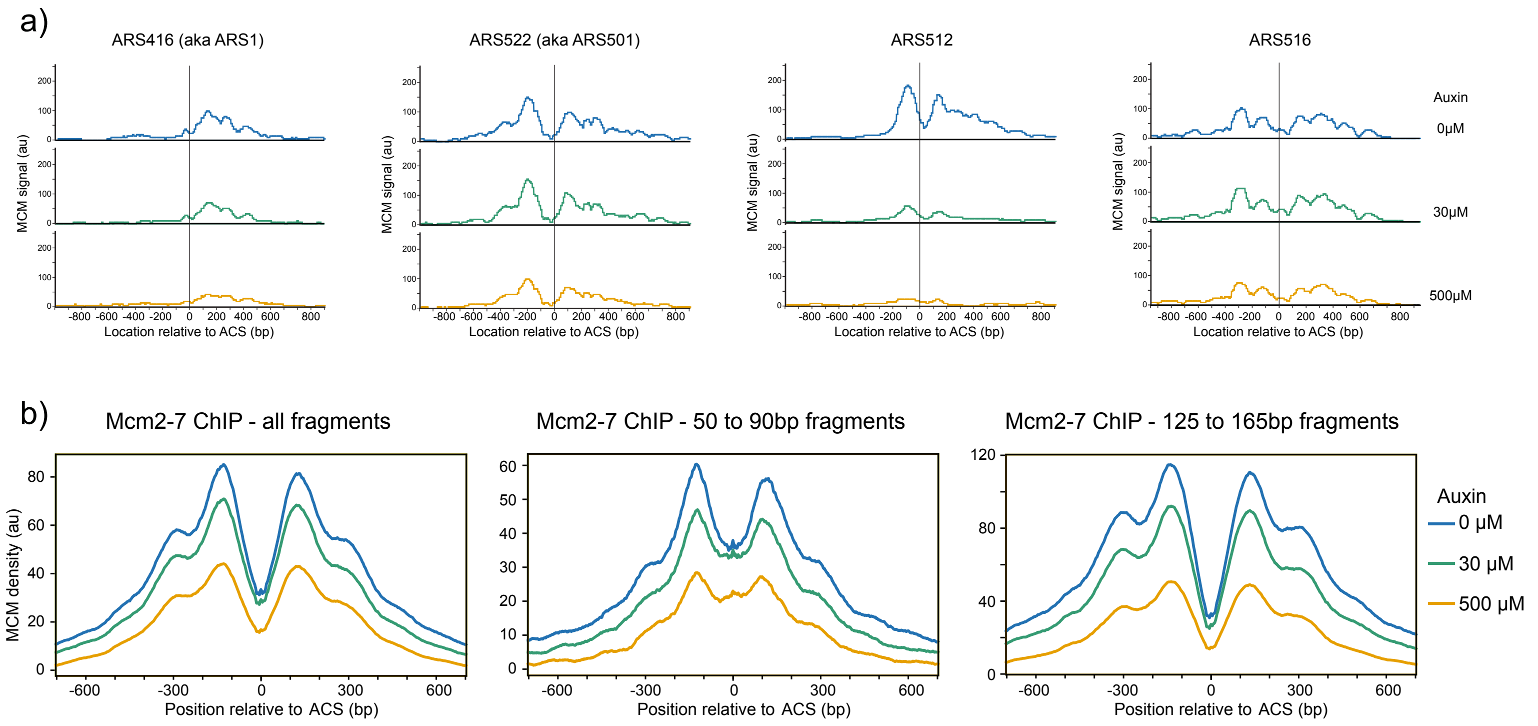

Supplemental Figure 5: Effects of MCM depletion on MCM loading at origins.
a) MCM signal at well-known and example single origins in response to auxin treatment (yFS1059 - Replicate 2). Signal at the well-known ARS1 and ARS501 origins of replication shows slight reductions in response to treatment with 0 μM, 30 μM, and 500 μM auxin. ARS512 loses most of its MCM signal in response to auxin treatment, while ARS516 signal remains largely the same.
b) Normalized MCM MNase-ChIP-seq density profiles centered at ACSs for the indicated auxin treatments (yFS1059 - Replicate 2) for all, MCM-sized (50-90 bp) or nucleosome-sized fragments (125-165 bp).
