# Supplementary material for: The capacity of origins to load MCM establishes replication timing patterns
Source: PLoS Genet. 2021 Mar 25;17(3):e1009467. doi: 10.1371/journal.pgen.1009467 (PMC8023499; doi:10.1371/journal.pgen.1009467)
Supplement: S6 Fig — Fragment Analyzer (Advanced Analytical Technologies) results of sequencing-ready input libraries show the different degrees of digestion for Replicates #1 and #2 of yFS1059. Replicate #2 displays higher mononucleosome-sized (146bp nuc-fragment + sequencing adapters and primers = ~284bp) content and therefore a stronger digestion. (PDF) [file pgen.1009467.s006.pdf]

# Supplemental Figure 6

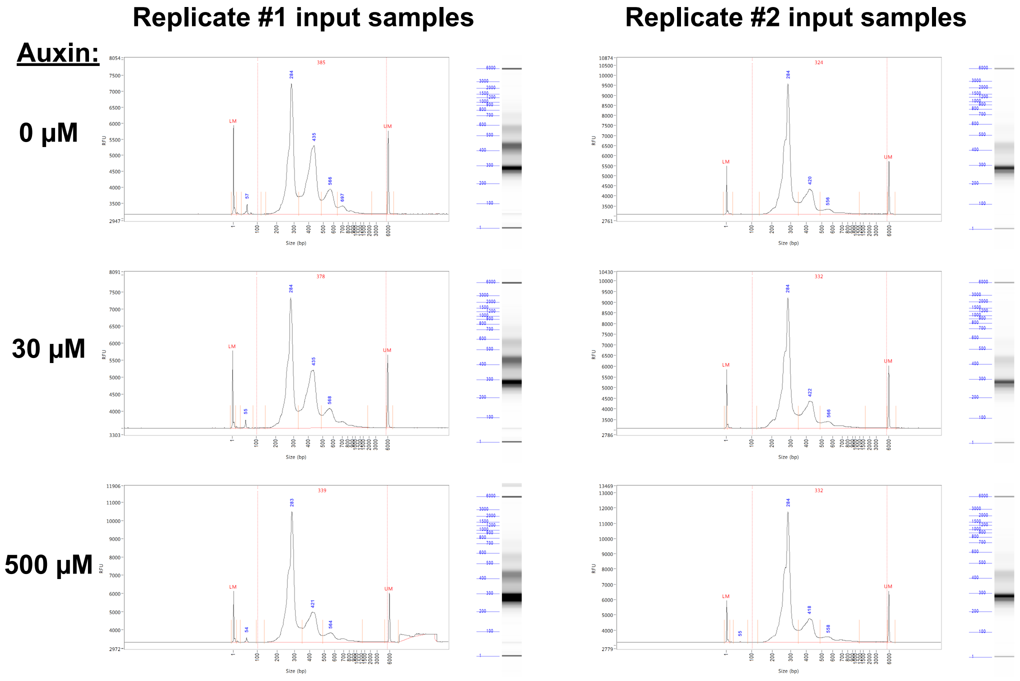


**Supplemental Figure 6: Fragment analyzer results for the two MCM reduction replicates shows different degrees of MNase digestion**

Fragment Analyzer (Advanced Analytical Technologies) results of sequencing-ready input libraries show the different degrees of digestion for Replicates #1 and #2 (yFS1059 strain). Replicate #2 displays higher mononucleosome-sized (146bp nucleosome-fragment + sequencing adapters and primers = ~284bp) content and therefore a stronger digestion.
