# Supplementary material for: The capacity of origins to load MCM establishes replication timing patterns
Source: PLoS Genet. 2021 Mar 25;17(3):e1009467. doi: 10.1371/journal.pgen.1009467 (PMC8023499; doi:10.1371/journal.pgen.1009467)
Supplement: S7 Fig — a) Input (non-IP) read coverage density profiles at ARS origins of replication for Replicates #1 and #2 of yFS1059 for the indicated auxin treatments. b) Comparison of input coverage within 1 kb of ARS origins between 0 μM versus 30 μM and 500 μM auxin treatments. (PDF) [file pgen.1009467.s007.pdf]

Supplemental Figure 7

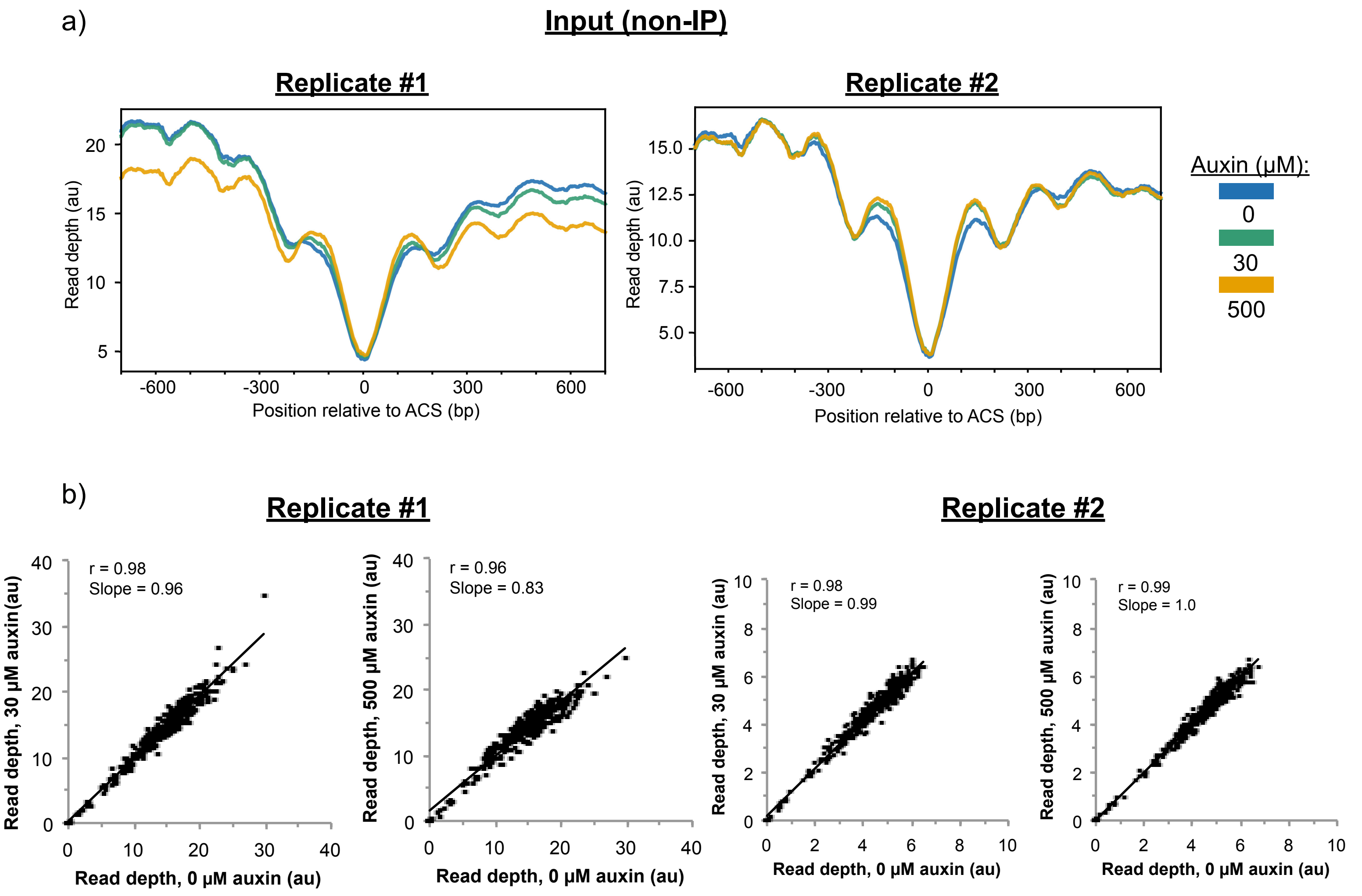

Supplemental Figure 7: Input density profiles and origin coverage quantitations do not change significantly after auxin-induced Mcm4 degradation

a) Input (non-IP) read coverage density profiles at ARS origins of replication for Replicates #1 and #2 for the indicated auxin treatments (yFS1059 strain).
b) Comparison of input coverage within 1 kb of ARS origins between 0 µM versus 30 µM and 500 µM auxin treatments.
