# Supplementary material for: The capacity of origins to load MCM establishes replication timing patterns
Source: PLoS Genet. 2021 Mar 25;17(3):e1009467. doi: 10.1371/journal.pgen.1009467 (PMC8023499; doi:10.1371/journal.pgen.1009467)
Supplement: S8 Fig — MCM ChIP-seq signal in yFS1059 was quantified 500 bp upstream and downstream of ACSs. Origins were separated based on the dominant signal. Heatplots were generated for the set of origins displaying higher MCM signal upstream of the ACS as well as for the set of origins displaying higher signal downstream of the ACS. (PDF) [file pgen.1009467.s008.pdf]

Supplemental Figure 8

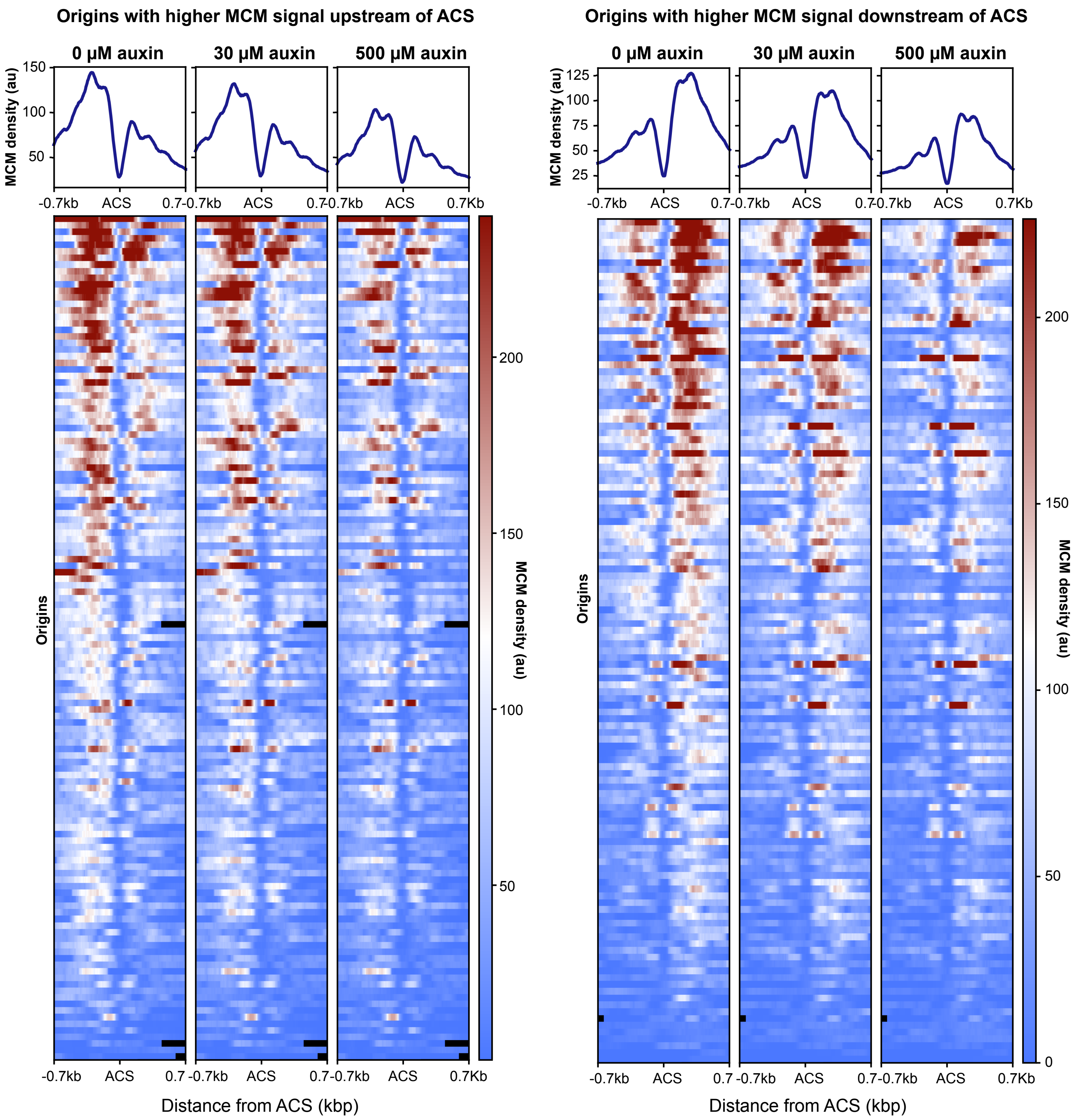

Supplemental Figure 8: ACS-annotated origins separated by magnitude of signal upstream or downstream of ACS

MCM ChIP-seq signal was quantified 500 bp upstream and downstream of ACSs (yFS1059 strain). Origins were separated based on the dominant signal. Heatplots were generated for the set of origins displaying higher MCM signal upstream of the ACS as well as for the set of origins displaying higher signal downstream of the ACS.
