# Supplementary material for: The capacity of origins to load MCM establishes replication timing patterns
Source: PLoS Genet. 2021 Mar 25;17(3):e1009467. doi: 10.1371/journal.pgen.1009467 (PMC8023499; doi:10.1371/journal.pgen.1009467)
Supplement: S9 Fig — Correlation between MCM signal and replication timing for origins that are known to be affected by specific replication timing control factors [20]. (PDF) [file pgen.1009467.s009.pdf]

Supplemental Figure 9

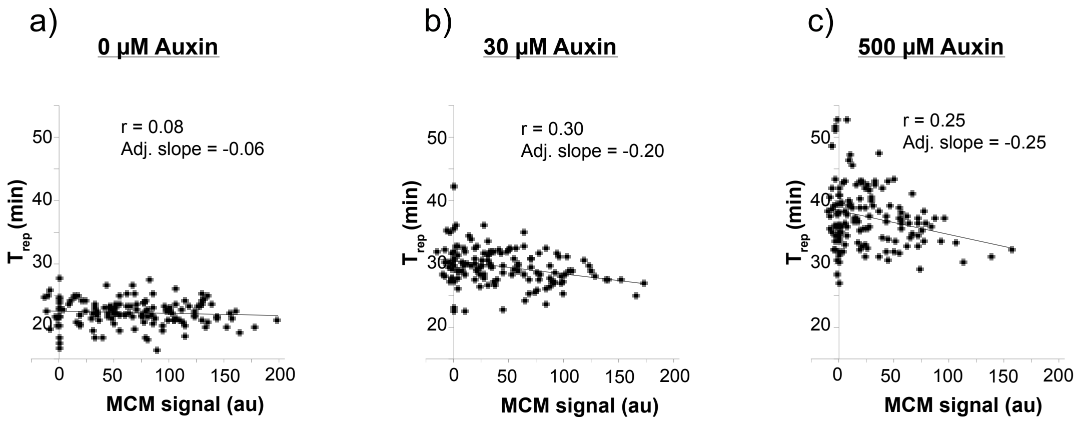

Supplemental Figure 9: Correlation of MCM signal and replication timing for origins that are affected by specific timing mechanisms

Correlation between MCM signal and replication timing for origins that are known to be affected by specific replication timing control factors (Das et al., 2015).
