# Supplementary material for: The capacity of origins to load MCM establishes replication timing patterns
Source: PLoS Genet. 2021 Mar 25;17(3):e1009467. doi: 10.1371/journal.pgen.1009467 (PMC8023499; doi:10.1371/journal.pgen.1009467)
Supplement: S10 Fig — a) Serial dilution assay of exponentially growing cultures of the indicated overexpressing genotypes, grown in the presence of raffinose or galactose. (-) = yFS1020, MCM2,3,4,5,6,7(GFP) = yFS1075; MCM2,3,4,5,6,7, CDT1 = yFS1021; CDT1 = yFS1080; MCM2,3,4,5,6,7 = yFS1076. b) Cultures of yFS1020, a control strain with no overexpression vectors, were treated as in Fig 5A and released into S phase. Flow cytometry quantitation shows the S phase progression of cells supplemented with glucose during the α-factor arrest versus those there were not. (PDF) [file pgen.1009467.s010.pdf]

Supplemental Figure 10

a)

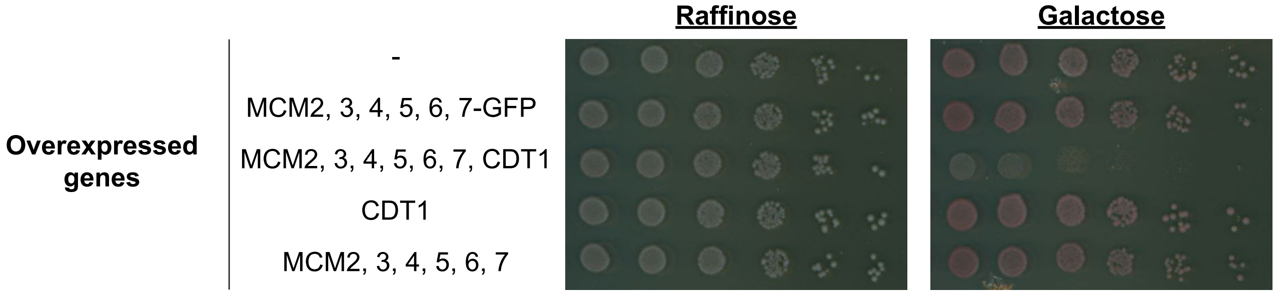

b)

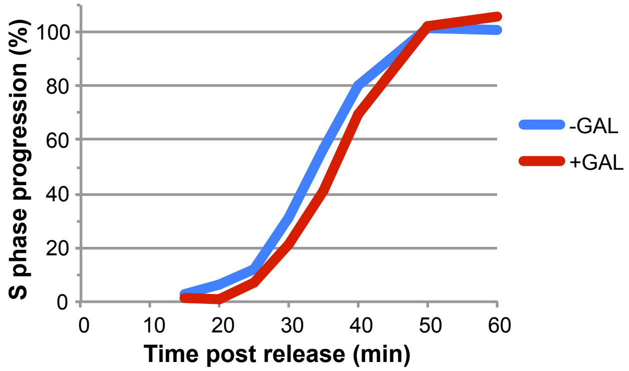

Supplemental Figure 10: MCM overexpression does not affect cell viability

a) Serial dilution assay of exponentially growing cultures of the indicated overexpressing genotypes, grown in the presence of raffinose or galactose. (-) = yFS1020, MCM2,3,4,5,6,7-GFP = yFS1075, MCM2,3,4,5,6,7, CDT1 = yFS1021, CDT1 = yFS1080, MCM2,3,4,5,6,7 = yFS1076.
b) Cultures from yFS1020, a control strain with no overexpression vectors, were treated as in **Figure 5a** and released into S phase. Flow cytometry quantitation shows the S phase progression of cells supplemented with glucose during the α-factor arrest versus those there were not.
