# Supplementary material for: The capacity of origins to load MCM establishes replication timing patterns
Source: PLoS Genet. 2021 Mar 25;17(3):e1009467. doi: 10.1371/journal.pgen.1009467 (PMC8023499; doi:10.1371/journal.pgen.1009467)
Supplement: S11 Fig — a) MCM ChIP-seq signal at ARS origins for the two main ‘untreated’ samples in this study: 0 μM auxin condition (Fig 1A–yFS1059) versus (-) galactose condition (Fig 5A–yFS1075). b) Comparison of MCM signal at origins between the (-) galactose condition in this study (yFS1075) and other publications [18,42]. c) Replication timing correlation between the (-) galactose condition (yFS1075) and: i) 0 μM auxin condition for ARS origins, and ii) genome-wide 1 kb windows from Mueller et al [59]. (PDF) [file pgen.1009467.s011.pdf]

Supplemental Figure 11

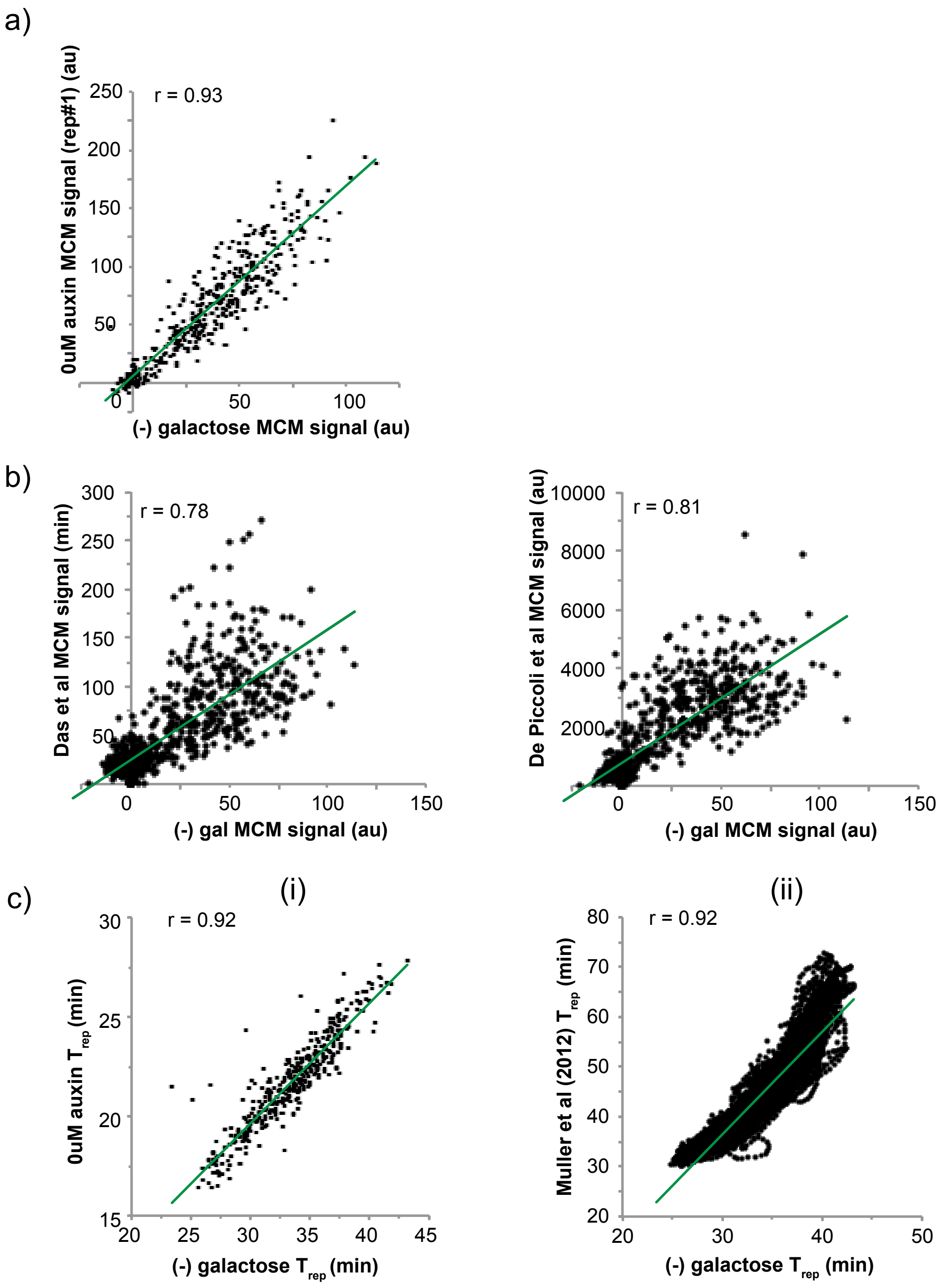

Supplemental Figure 11: MNase-ChIP-seq and replication timing results from the MCM overexpression experiments are reproducible

a) MCM ChIP-seq signal at ARS origins for the two main 'untreated' samples in this study: 0 μM auxin condition (**Figure 1a** – yFS1059) versus (-) galactose condition (**Figure 5a** – yFS1075).
b) Comparison of MCM signal at origins between the (-) galactose condition in this study (yFS1075 strain) and other publications (Das et al., 2016, DePicoli et al., 2012).
c) Replication timing correlation between the (-) galactose condition (yFS1075 strain) and: i) 0 μM auxin condition for ARS origins, and ii) genome-wide 1 kb windows from Mueller et al, 2012.
