# Supplementary material for: The capacity of origins to load MCM establishes replication timing patterns
Source: PLoS Genet. 2021 Mar 25;17(3):e1009467. doi: 10.1371/journal.pgen.1009467 (PMC8023499; doi:10.1371/journal.pgen.1009467)
Supplement: S12 Fig — a) Quantitation of MCM signal at ARS origins when MCM is overexpressed as in Fig 5A but for 3 hours (yFS1075). b) Quantitation of MCM signal at ARS origins for a control strain that does not overexpress any genes (yFS1020). c) Quantitation of MCM signal and Trep at ARS origins when MCM is overexpressed along with Cdt1 as in Fig 5A (yFS1021). (PDF) [file pgen.1009467.s012.pdf]

Supplemental Figure 12

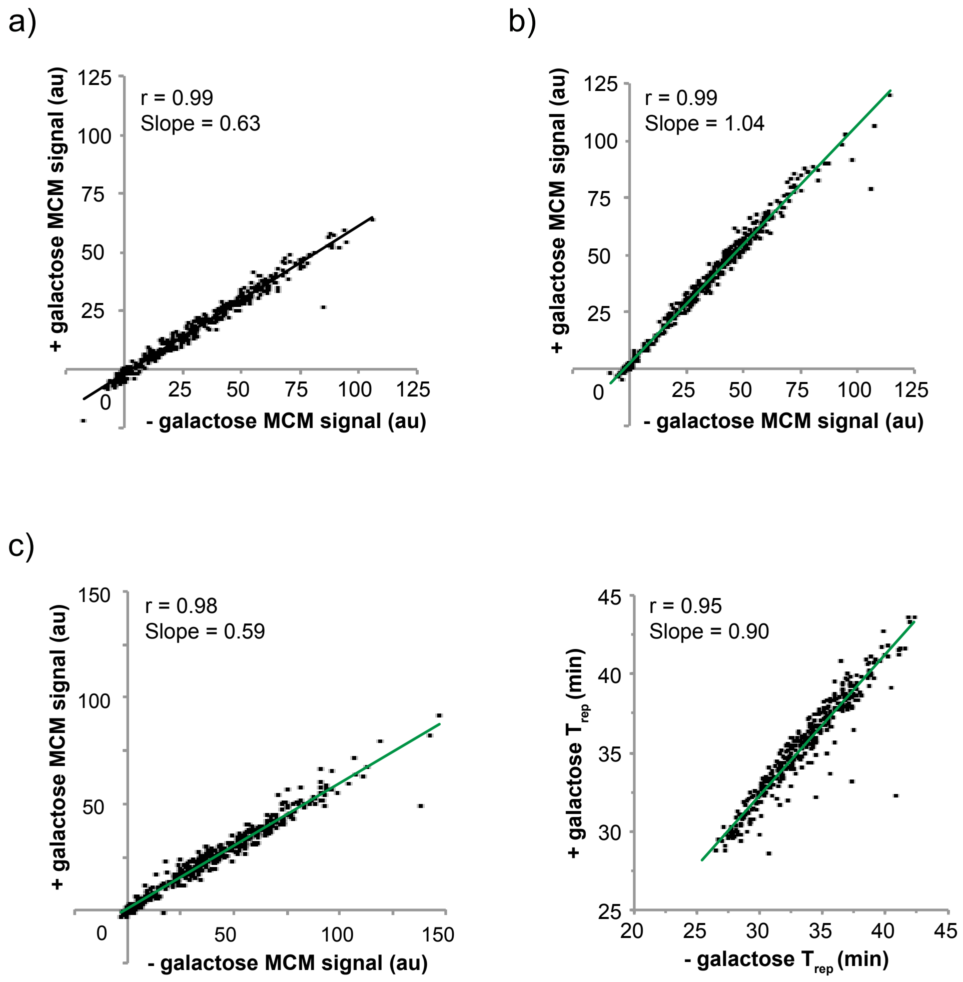

Supplemental Figure 12: Overexpression of MCM for longer times or along with Cdt1 does not alter loading levels at origins or replication timing

a) Quantitation of MCM signal at ARS origins when MCM is overexpressed as in **Figure 5a** but for 3 hours (yFS1075).
b) Quantitation of MCM signal at ARS origins for a control strain that does not overexpress any genes (yFS1020).
c) Quantitation of MCM signal and T_{rep} at ARS origins when MCM is overexpressed along with Cdt1 as in **Figure 5a** (yFS1021).
