# Supplementary material for: The capacity of origins to load MCM establishes replication timing patterns
Source: PLoS Genet. 2021 Mar 25;17(3):e1009467. doi: 10.1371/journal.pgen.1009467 (PMC8023499; doi:10.1371/journal.pgen.1009467)
Supplement: S13 Fig — WT (yFS1020), MCM7-GFP (yFS1083), MCM2, 3, 4, 5, 6, 7-GFP overexpressing (yFS1075), and MCM2, 3, 4-GFP, 5, 6, 7 and CDT1 overexpressing (yFS10810) cultures were grown as in Fig 5A in the presence of galactose and visualized at the α factor arrest time point using DAPI to stain DNA and GFP fluorescence to visualize MCM quantity and localization. (PDF) [file pgen.1009467.s013.pdf]

Supplemental Figure 13

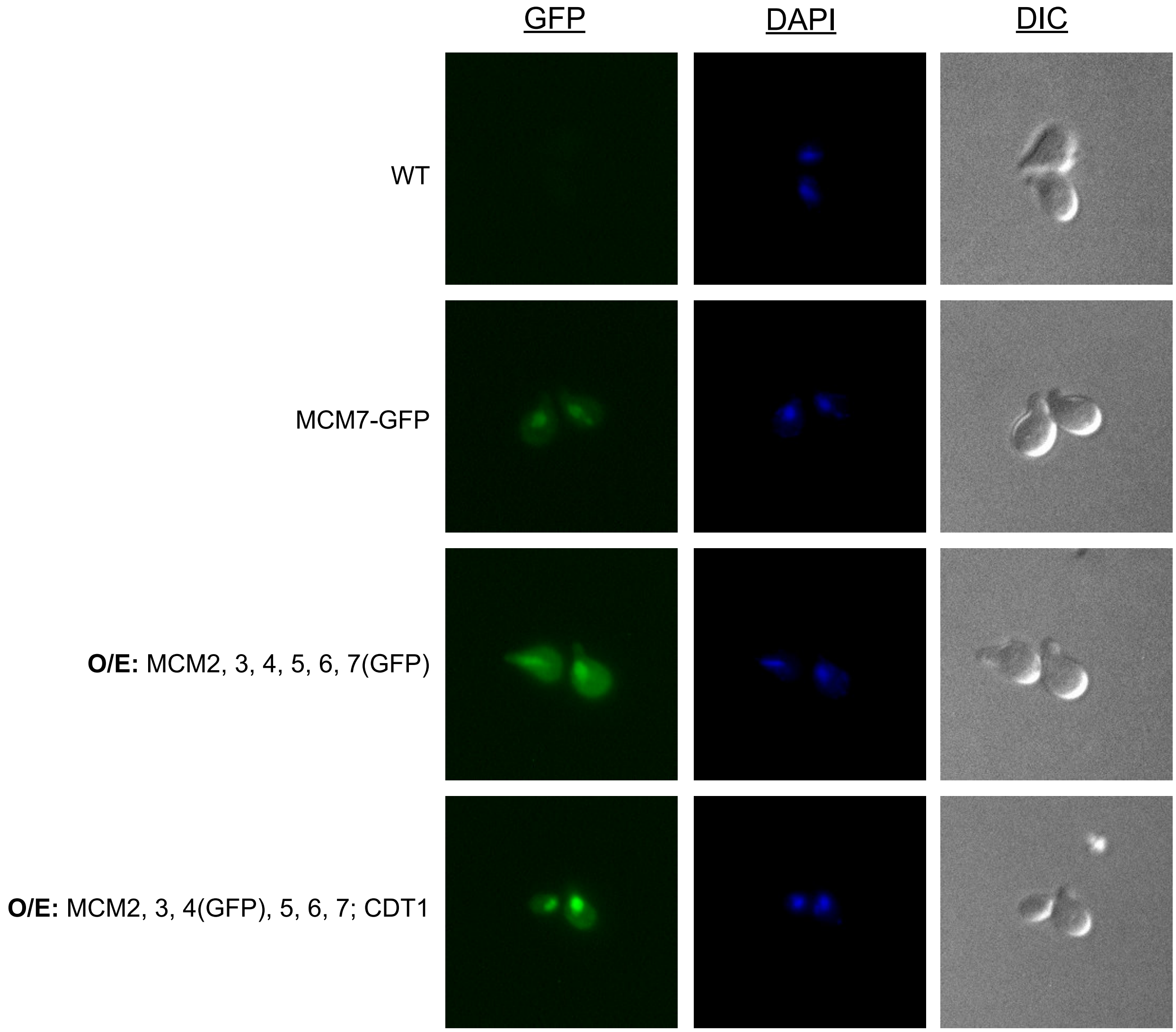

Supplemental Figure 13: Overexpressed MCM is able to enter the nucleus during α-factor arrest

WT (yFS1020), MCM7-GFP (yFS1083), MCM2, 3, 4, 5, 6, 7-GFP overexpressing (yFS1075) and MCM2, 3, 4-GFP, 5, 6, 7 and CDT1 overexpressing (yFS10810 cultures were grown as in **Figure 5a** in the presence of galactose and visualized at the α factor arrest time point using DAPI to stain DNA and GFP fluorescence to visualize MCM quantity and localization.
