# Supplementary material for: The capacity of origins to load MCM establishes replication timing patterns
Source: PLoS Genet. 2021 Mar 25;17(3):e1009467. doi: 10.1371/journal.pgen.1009467 (PMC8023499; doi:10.1371/journal.pgen.1009467)
Supplement: Attachment — Submitted filename: 110765_1_rebuttal_1926649_qkd55y.doc [file pgen.1009467.s019.doc]

Reviewer #1 (Evidence, reproducibility and clarity):

This manuscript follows on from previous work from the Rhind lab to investigate whether the load of MCMs at origins is a factor in when the origin activate (as a population average) during S phase. The authors use budding yeast and a auxin degron system to modulate the levels of an MCM subunit. This allows them to titrate down the concentration of the MCM hexamer and observe the effect. Crucially, they assay both the reduction in MCM load at origins and the subsequent replication dynamics in the same experiment. This is the power of their approach and allows them to rigorously test their hypothesis.

**Major comments**

1.I found the introductory paragraph discussing the Rhind lab hypothesis about the possibility of multiple MCM being loaded at origins somewhat misleading. The first paragraph of the discussion was much clear. However, I feel that the introductory paragraph should deal with the difference between the two proposals: 0-1 MCM-DH per origin (de Moura et al), vs 0-50+ MCM-DH (Yang et al). It s also important to note that Foss et al find that "In budding yeast, [MCM] complexes were present in sharp peaks comprised largely of single double-hexamers" - i.e. consistent with 0-1 MCM-DH per origin.

To improve the balance of the introduction, I think the authors should briefly introduce the concepts behind the 0-1 MCM-DH per origin; this was defined as origin competence by Stillman and clearly described by McCune et al (2008; see figure 8) prior to the work from de Moura et al.

Furthermore, in the discussion the authors should be more even-handed. To date there is no data to conclusively rule one way or the other in distinguishing between single vs multiple MCMs. The authors cite Lynch et al and state "overexpression of origin-activating factors in S phase causes most all origins to fire early in S phase, consistent with most origins having at least one MCM loaded". However, Lynch et al report equivalent (roughly equal) origin efficiencies, but the assay doesn't distinguish between all going up to high efficiency or all going to a lower intermediary efficiency. Given that fork factors (polymerases, etc) are likely to become limiting at some point (or checkpoints could be activated due to limited dNTP supplies) it would seem plausible that uniform origin efficiency could be a consequence of less than maximal origin firing. As part of this discussion it would be useful for the authors to include what conclusions have been reached on MCM load from in vitro systems (with chromatin substrates).

Because the main focus of the paper is not dependent on whether MCM stoichiometry varies from 0 to 1 or 0 to many, we had relegated our discussion of absolute stoichiometry to the Discussion. However, it is clear from multiple reviewer's comments that it is something very much on readers minds. Therefore, we have now included a brief introduction to the 0-to-1 and 0-to-many scenarios in the Introduction and moved the bulk of the discussion of the data supporting the two scenarios to the Discussion.

2.The authors are not the first to look at the consequence of reduced MCM concentrations on origin function. This was essentially the basis for the MCM screen undertaken by Bik Tye's lab that first identified the MCM genes. In addition to temperature sensitive mutants, the Tye group also examined heterozygotes (Lei et al., 1996) to show differential effect on the ability of two origins to support plasmid replication. The authors finds are entirely consistent with these early studies, particularly since ARS416 (formerly ARS1) was found to highly sensitive to reduced MCM levels and ARS1021 (formerly ARS121) was found to be insensitive to MCM levels. The authors find a signifiant reduction in MCM load at ARS416, but the MCM load at ARS1021 is unaltered by reduced MCM concentration. It would be worth the authors noting this consistency. The authors do cite the Lei study, but not in this context. The original MCM screen was published here:

Maine, G., Sinha, P., Tye, B. (1984). Mutants of S. cerevisiae defective in the maintenance of minichromosomes Genetics 106(3), 365 - 385.

Furthermore, at the end of the discussion the authors state that "it will be interesting to dissect the specific cis- and trans-acting factors that make origins sensitive or resistant to changes in MCM levels". The equivalent effect reported by the Tye lab has already been dissected by the Donaldson lab (Nieduszynski et al., 2006) and perhaps it would be worth briefly mentioning their findings.

We have included both of these literature precedents in the Discussion.

3.The authors should show the flow cytometry data for each of their cell cycle experiments, if only in supplementary figures. This is important to allow a reader (and reviewer) to judge the level of synchrony achieved when interpreting the results.

This data is now included as Figure S1

4.I think the authors should show the ChIP signal at some example origins, including ones sensitive and insensitive to the reduction in MCM concentration. Currently all the high resolution ChIP data (i.e. over 1400 bp, e.g. Fig 3a) is presented as meta-analyses of many origins.

We will include this analysis in a subsequent revision.

5.When describing the results in Fig 4a the authors focus on changes (highlighted in black boxes) that fit their expectation. However, there are other sites that should at least be mentioned that don't seem to fit the authors model, e.g. ARS517, ARS518. It would be worth discussing what fraction of the timing data can be explained by the reduced MCM load.

We now explicitly point out that Figures 4c and 4d address this issue of the robustness of the correlation. Although there is significant variation, as the reviewer points out, the trend is seen genome wide. As it happens, both ARS517 and ARS518 do fit the model reasonably well. They have intermediate loss of MCM signal and intermediate delay in timing.

**Minor comments**

-These data, rather than this data (throughout).

I suspect that the journal style and/or copy editors will make the final call. However, I will point out that although 'data' is most certainly plural in Latin, its predominate modern English usage is as a mass noun, such as water or sand or information. In general, users do not think of, or use, 'data' as a collection of discrete elements, each on being a 'datum', a contention supported by the very infrequent use of the word datum. For instance, in ChIP-seq experiment, what is a datum? Each individual read? Each individual nucleotide in each read? The quality score for each individual nucleotide in each read? Each pixel in each image from the sequencer? When one wants to refer to an individual piece of data, common usage is to refer to a data point, just as one would refer to a grain of sand. Moreover, if 'data' were plural, it would be incorrect to use it in phrases such as "there is very little data available". Would the review really suggest using "there are very few data available"?

-the authors should clearly state in figure legends what window size has been used in analysing genomic data.

All analyses were done using 1kb windows, as now stated in the figure legends.

-in figure 2a the authors show pairwise comparisons between conditions, it would be nice to see the 3rd pairwise comparisons perhaps as a supplementary figure

We have included the third comparison in Figure 2a.

-in figure 2c it would be clearer to use the same colour for the lines and the points

The regression lines are in the same colors as the data points they fit. x=y is shown in blue for comparison, as now noted in the figure legend.

-the authors should avoid the use of red/green colour combinations in their figures (see: https://thenode.biologists.com/data-visualization-with-flying-colors/research/)

All figures will be redrawn in colorblind-accessible colors in a subsequent revision.

-in the text the authors state "ORC binding to the ACS and subsequent MCM loading is a directional process dependent on a ACS- site and a similar but inverted nearby sequence (Xu et al., 2006)". I think it would be more appropriate to cite the following study here:

Coster, G., Diffley, J. (2017). Bidirectional eukaryotic DNA replication is established by quasi-symmetrical helicase loading Science (New York, NY) 357(6348), 314 - 318. https://dx.doi.org/10.1126/science.aan0063

The Coster reference has been included.

-the list of factors that influence replication timing should include Rif1, whereas it is less clear that Rpd3 acts within the unique genome (as opposed to indirectly via repetitive DNA, e.g. rDNA)

Rif1 has been added to the list.

-figure 4 - it might help to mark the centromere on panel a. Also, why do the ChIP peaks and annotated origins appear to line up so poorly?

The shift between the peaks and the ACS positions was introduced during the construction of the figure. Thanks for catching it. The alignment has been corrected and the centromere annotation has been added.

-figure 4d - would it not be better to use fraction of lost MCM signal on the x-axis as in previous figures?

If T_rep was a linear function of MCM stoichiometry, fraction lost would work as well as amount lost. However, we find that there is a lower correlation between fraction of MCM signal lost and T_rep delay than between absolute MCM signal lost and T_rep delay, suggesting a more complicated relationship.

-"with galactose or raffinose, to induce or repress Mcm2-7 overexpression, respectively." This is incorrect, raffinose does not repress this promoter (that requires glucose).

Fixed.

-the S. pombe spike in is a great addition to the over expression experiments. It's a shame that it wasn't included in the auxin experiments.

Yes, we agree.

-why does the data in fig 5d appear to be at much lower resolution that the previous ChIP data?

The resolution was inadvertently reduced during the rendering of the figure. The resolution has restored.

-in the sequencing analysis pipeline for MCM ChIP the authors use a 650 bp upper size limit; why have such a large threshold compared to the size of a nucleosome? Are the analyses and findings sensitive to this size threshold?

Although the MNase digestion was optimized to produce mostly mononucleosomal-sized digestion, some di- and very little tri- nucleosomal fragments still remain. In order to capture as many of the MCM-protected immunoprecipitated fragments as possible, the upper limit was set at 650 bp (up to 4 nucleosomes-worth of DNA). However, there is a very minimal contribution from fragments larger than mononucleosomes, qualitatively as well as quantitatively in 1kb windows around origins. Figure 3a provides a qualitative depiction of the contribution of dinucleosomes (input, ~300bp).

-the repliscope package was published here:

Batrakou, D., Müller, C., Wilson, R., Nieduszynski, C. (2020). DNA copy-number measurement of genome replication dynamics by high-throughput sequencing: the sort-seq, sync-seq and MFA-seq family. Nature Protocols 15(3), 1255 - 1284. https://dx.doi.org/10.1038/s41596-019-0287-7

The reference has been corrected.

Reviewer #1 (Significance):

This work builds upon a body of work from the Rhind group (and others) to determine the contribution of MCM load to replication origin activation dynamics. To my mind this is the most convincing dataset and analysis to date and goes a long way to supporting the model that the efficiency of MCM loading is a major factor in determining the mean replication time of an origin. As the authors state, they are still not able to distinguish between two different models of MCM load (single vs multiple). It would be interesting for the authors to discuss how these two models could be distinguished in the future (perhaps with single cell/molecule experiments).

This study will be of interest to those in the fields of DNA replication and genome stability.

My field of expertise is DNA replication and replication origin function.

Reviewer #2 (Evidence, reproducibility and clarity):

**Summary:**

This is a nice study that characterizes the consequences of limiting or increasing Mcm expression on the replication program. Prior ChIP experiments in yeast have observed that not all origins exhibit the same level of Mcm enrichment and that increased mcm enrichment was correlated with origin activity. These observations led to two different models -- a) that multiple Mcm2-7 double hexamer complexes are loaded at some origins and b) a probabilistic model where the differential enrichment of Mcm2-7 reflected the fraction of cells in a population that had loaded the Mcm2-7 complex at a specific origin. While the titration experiments presented here don't provide any conclusive support for either model, they do provide some novel and relevant insights for the replication field, in part, due to the increased resolution and quantification afforded by the MNase ChIP-seq approach (and S. pombe spike in). The authors very nicely demonstrate that origins are differentially sensitive to Mcm2-7 depletion and that loss of Mcm2-7 loading results in an altered replication timing profile. The origins most impacted by loss of Mcm2-7 are 'weak' origins as described by the Fox group. Intriguingly, the authors find that the 5X overexpression of Mcm2-7 does not perturb the relative Mcm2-7 loading at individual origins, but rather instead globally represses Mcm2-7 association at all origins. They also find that overexpression of both Cdt1 and Mcm2-7 is detrimental to the cell (although no obvious replication phenotype was observed). Finally, the authors present a reasonable interpretation of their data in the context of models for replication timing which was very well articulated.

**Major Comments:**

From the methods it appears that different analyses were performed with different replicates?

"Replicate #1 was used for all analyses except for V plots, for which the higher resolution Replicate #2 was used."

Ideally all of the conclusions should be supported by all the replicates independently, or if the replicates are concordant -- they should be merged (at a similar sequencing depth) prior to doing the analyses.. Even the v-plots with merged replicates will be informative due to the greater sequencing depth.

Though we agree that greater sequencing depth would be informative for aggregation analysis, we think that one of the main strengths of our study is the analysis of MCM quantitation and replication timing in the same population of cells. Although the experiments were performed in exactly the same way, there is always slight biological or temporal differences between the replicates, due to the complicated nature of the experimental design. This variation increases the noise between the MCM ChIP and the replication timing analyses. Therefore, were analyzed the replicates separately. However, we did do all of the analyses on both replicates and got similar results. We have now explicitly stated as much.

The authors should provide a separate analysis for the larger nucleosomal sized fragments and smaller putative MCM double hexamer fragments with regards to the Mcm loading and relationship to ACS and orientation. They may represent an interesting intermediate with mechanistic consequences for the interpretation.

We will include the suggested analysis in a subsequent revision.

The authors should present the v-plots and an analysis of which side the Mcm's load for the overexpression studies. I was surprised that there was no further in-depth analysis for these two extremes. Perhaps similar conclusions will be reached, but it should at least be mentioned/presented as a supplementary figure.

We will include the suggested analysis in a subsequent revision.

**Minor Comments:**

This is largely semantic, but the majority of MNase ChIP-seq signal recovered is associated with the nucleosomes and not in the NDR and as the signal in the NDR is differentially sensitive to digestion, I would suggest rephrasing the following sentence:

"In contrast to previous genome-wide reports (Belsky et al., 2015), but in agreement with recent in-vitro cryo-EM structures (Miller et al., 2019), we also observe MCM signal in the nucleosome-depleted region (NDR) of origins. "

to :

"In agreement with a previous genome-wide report (belsky 2015), we found that the bulk of the MCM signal was associated with nucleosomal sized fragments; however the increased resolution afforded by our approach allowed us to also detect protected fragments in the NDR as predicted by recent in vitro cryo em structures..."

We have modified the sentence as suggested.

As a sanity check, please double check V-plots and presence of small fragments with the digestion conditions. In the Henikoff manuscript the bulk of sub-nucleosomal fragments were lost with the longer digestion time. Specifically, the TF footprints were more pronounced with minimal digestion. While it might be argued that the longer digestion more tightly resolved the binding site, in many cases they were completely lost with the 20 minute digestion. This is just a simple check -- I don't doubt the results as reported given the experimental conditions are very different. For example, the henikoff manuscript did not use cross linking or an antibody enrichment step.

We double checked and confirmed that more small fragments are found in the more digested library. The reason that we see more small fragments when we digest more, in contrast to the contrary observation in the Henikoff paper is presumably because MCM has a larger footprint than a transcription factor and protects that footprint more effectively.

Last paragraph of the "MCM associates with nucleosomes section" which reports that the Mcm2-7 complex is loaded up or downstream from the ACS independent of orientation should cite Belsky 2015 (Figure 5 and discussion) for the initial observation.

Done.

The authors argue that the global reduction in MCM loading associated with overexpression may be a technical artifact given that all origins exhibit a proportional reduction in mcm2-7 loading. However, this is exactly what the S. pombe spike in control is intended for. The relative difference between individual origins resulting from Mcm2-7 depletion would still be evident without the spike in. The authors do discuss different possibilities, but I would not be so keen to discard this as technical artifact.

We, too, are reluctant to dismiss this result as a technical artifact. However, we are at a loss to offer any other explanation. We raise a handful of biological possibilities in the Discussion, but dismiss each one as failing to account for our results. We would be happy to entertain other suggestions.

Reviewer #2 (Significance):

This work has several advances that will be appreciated by the replication field -- including a high resolution view of Mcm2-7 loading in the context of chromatin; the impact of titrating (low and high) MCM expression on MCM loading and replication timing program; and a well reasoned discussion of how different models of MCM loading would impact origin activation and replication timing program. The work builds on prior studies in the field (eg. Belsky 2015), while some of the conclusions regarding the localization of the Mcm2-7 complex relative to the ACS and surrounding nucleosomes are confirmatory, the increased resolution provides new insight (like the enrichment of small fragments in the NDR) that could be further strengthened by additional analysis (see above).

My expertise is DNA replication and chromatin.

Reviewer #3 (Evidence, reproducibility and clarity (Required)):

In this study, the authors use Auxin-mediated degradation of Mcm4 to reduce the concentration of the MCM helicase complex in yeast, and determine the effects of this reduction on both MCM-origin association (interpreted as MCM loading) by MNase-MCM-ChIPSeq and on replication origin function by Sync-Seq replication timing experiments (deep sequencing of a yeast population as it progresses through a synchronized S-phase). Complementary experiments testing the effect of induced MCM complex over-expression on MCM-origin association are also performed.

The authors find that reducing Mcm4 levels (and thus loading-competent MCM complexes) causes yeast cells to be more sensitive to DNA replication stress. In addition, not all origins are equally susceptible to reductions in MCM levels; the origins that do lose MCM binding at reduced MCM levels show a reduction in activity and an associated delay in their replication time under those conditions. Finally, over-expression of the MCM complex has no effect on MCM-origin association or origin function, suggesting that MCM levels are not limiting for origin licensing in yeast under normal lab conditions. The strengths of the study are the well-executed experiments and very nice data that are presented. However, there are several weaknesses. The authors make conclusions that are not supported by their data; and several of the outcomes are not at all unexpected based on extensive published studies in yeast and mammalian cells, raising issues about whether this study advances and/or clarifies the current gaps in the field. While some of the relevant past studies were referenced, the authors did not place their own study in the context to published work and current models in the field, which reduced the scholarly value of their study. Because the work was not placed in context of the field, some of the rationale and conclusions were misleading.

**Some specific major comments:**

1,The title is misleading. The authors have clearly shown that when MCM levels are be made limiting in an engineered system, some origins are substantially less active, which means that these origin loci are replicated "passively" (i.e. by a Replication Fork (RF) emanating from a distal origin) rather than actively (i.e. by "firing" and initiating replication). Their own replication data show that. But this competition is only revealed when MCM levels are artificially/experimentally lowered. What is the evidence that competition for MCM complexes among individual origins establishes replication timing patterns in yeast? If anything, the over-expression experiment suggests the opposite--that MCM levels are not limiting and therefore do not play a substantial role in establishing the replication timing patterns that are observed in yeast. Instead those patterns appear to result primarily from the fact that MCM complex activation factors are present in limiting concentrations relative to origins.

We agree with the reviewer's analysis and have revised the title to "The Capacity of Origins to Load MCM Establishes Replication Timing Patterns".

2,The abstract states that "the number of MCMs loaded onto origins has been proposed to be a key determinant of when those origins initiate DNA replication during S-phase". While it is true that this lab has proposed this model in budding yeast, the current study performs no experiments that directly address this model--i.e. that i. individual origins possess a different number of MCM complexes and or ii that these differences underlie timing differences. They acknowledge this point in their Discussion--a ChIPSeq experiment is an ensemble experiment--there is no way to know that differences in MCM signals correspond to a different number of MCM complexes per origin versus a differences in the fraction of cells that contain and MCM complex at all at a given origin . But this statement in the abstract, combined with their conclusion in the same section of the paper: "Our results support a model in which the loading activity of origins, controlled by their ability to recruit ORC and compete for MCM, determines the number of helicases

loaded, which in turn affects replication timing" implies that they have tested a model that they have not tested. Given how quickly readers "skim" the literature these days, a misleading abstract can do a lot of damage to a field. The results presented in this study neither support nor refute the model for the number of helicases loaded per origin, and the fact that reducing origin licensing efficiency by making the major substrate limiting reduces the number of licensed origins in a cell population is fully expected based on the current state of the field .

Four questions are addressed in this comment. The first is whether there is variable MCM stoichiometry at origins. The second is whether that variation ranges from 0 to 1 and 0 to many. The third is if the variation is stoichiometry affects replication timing. The fourth is how this variation in stoichiometry comes about.

Our work is based on the conclusion, supported by a substantial body of literature, that MCM loading stoichiometry varies among origins. Our data in this paper further supports this conclusion.

As the reviewer notes, and as we had tried to make clear, the data is this paper does not address the range of the variation. Moreover, as we also tried to make clear, our hypotheses, results and conclusions are not affected by whether the range is 0 to 1 or 0 to many.

This paper focuses on Questions 3 and 4. We have reworked the introduction to make these distinctions more clear.

We have also corrected the abstract to refer to "the stoichiometry", instead of "the number", of MCMs.

3,The rationale for the study as stated in the Introduction: "Although the molecular biochemistry of initiation at individual origins continues to be elucidated in great detail (Bleichert, 2019), the mechanism governing the time at which different regions of the genome replicate has remained largely elusive (Boos and Ferreira, 2019)." Is also misleading. In fact, in budding yeast (and other organisms) there have been several advances in this area particularly with respect to DNA replication origin activation. The S-phase origin activation factors are limiting for origin function, and factors such as Ctf19 at centromeres and Fkh1/2 at non-centromeric early-acting origins help to directly recruit the limiting S-phase factor, Dbf4, to origins. It is misleading to ignore this substantial progress and not make an effort to place this current study, which is important and one of the first to look directly at MCM loading control in yeast, into a relevant context with respect to what is known. What's interesting is that this S-phase model assumes/requires that most origins are, in fact, licensed and thus that differences in licensing efficiency are not a major driving of replication timing patterns in yeast. But we do not know why there are only subtle differences in MCM loading---this study may help explain that.

We have broadened the scope of our Introduction and Discussion to address these points. However, it is not the case that "there are only subtle differences in MCM loading". MCM ChIP-seq (<https://www.ncbi.nlm.nih.gov/pubmed/20351051>, <https://www.ncbi.nlm.nih.gov/pubmed/26359232> and this paper) and MCM ChEC-seq <http://dx.doi.org/10.1101/737742> both show well over ten-fold variation in MCM stoichiometry at origins. We have now explicitly made this point in the Introduction.

4,The authors link the differential ability of MCM loading deficiencies when MCM is made limiting to differences in ORC binding categories. The "weak" origins, that presumably bind ORC weakly, were most affected by reductions in MCM. Are these origins less efficient than the other categories, DNA and chromatin-dependent (using the origin efficiency metric data from the Whitehouse lab) where MCM binding is not reduced as much? In normal cells are these early or late origins? Is the idea that the role of excess MCM is to achieve a sufficient number or "back up" origins per cell to deal with potential stress, as proposed by the Blow and Schwob labs in tissue culture cells many years ago? It seems likely that the data reported here are in fact confirmations of those early studies in mammalian cells---which is useful to know even if not unexpected.

We will include the suggested analyses in a subsequent revision.

Excess MCM do, as has been long appreciated and as we discuss, contribute to replication-stress tolerance. However, that is not a major point of our paper.

5,Aren't the results that losing MCM signal corresponds to loss of origin activity peaks entirely expected? The same result would be obtained if you made a point mutation in that origin's ACS. Of course preventing an origin from being licensed will delay that region's replication time in S-phase because it now must be replicated passively. Licensing affects replication timing patterns because the MCM complex is the substrate for limiting S-phase factors, but that is far different from concluding that the number of MCMs at an origin is what controls the time in S-phase when an origin is activated.

Yes, "the results that losing MCM signal corresponds to loss of origin activity peaks [are] entirely expected". However, this is not the important result. The key result is that the distribution of MCM at origins is not uniformly affected, which leads to our conclusions that, in wild-type cells, origin capacity dominates MCM stoichiometry and that, when MCM become limiting, origin activity (probably determined by ORC affinity) becomes critical—neither of which were expected results. In any case, the expected correlation between MCM loading and origin activity was observed as a consequence of measuring MCM stoichiometry and replication timing and is an obvious analysis to include, so we did so.

6,The authors stated that the measured MCM abundance for the 43% of origins that are not known to be controlled by the multiple mechanisms that have been shown to control origin replication time. Is this because they think that MCM loading contributes to the timing control of only these origins? Was MCM loading not affected at any of these other origins when MCM levels were reduced? Are those 43% of origins in the "weak" binding category in terms of ORC? The rationale for eliminating so many origins from these analyses were not clear.

We propose that the probability of origin activation is the product of the stoichiometry of MCM at the origin and the rate of MCM activation, which may be affected by *trans*-acting factors. For the 43% of origins for which there is no known *trans*-acting regulation, the correlation with stoichiometry is stronger. However, the correlation holds when looking at all origin, too. The suggestion to look at only the 57% of origins with known *trans*-action regulation is a good one. We will include this analysis and the other suggested analyses in a subsequent revision.

7,Doesn't the data in Figure 4c at 0 mM auxin support the conclusion that differences in MCM ChIP signals have negligible effects on origin activation time, in contrast to the publication by Das, 2015 from this lab? Or is the point that these origins are sensitive to reductions in MCM levels and the more sensitive they are the more delayed their replication time (but again, doesn't that have to be true? If they are losing MCM signals they cannot function as origins, so they are replicated passively and, by definition, will show delayed replication timing. An origin is defined as such by a loaded MCM complex.)

No. The reason the correlation in 4c is not a good as in our previous work is that in Das 2015 we compared origin-activation efficiency (calculated from our stochastic model in Yang 2010), instead of T_rep, which we used here. T_rep is a convolution of origin-activation time and passive-replication time, reducing to correlation. The important observation is that the correlation gets better as MCM levels are reduced.

The correlation between MCM stoichiometry and activation efficiency may seem trivial, but just because a model is simple does not mean it is not correct. If stoichiometry was the only factor regulating origin activation, we would expect a stronger correlation. So, we conclude that there are other factors at play, quite possible the trans-acting factors that the reviewer mentions in their second point. However, if stoichiometry played no role, we would expect no correlation. So, we propose that MCM stoichiometry is "an important determinant of replication timing".

8,I do not understand the conclusions from Figure 4d. There is an extremely small positive correlation between how much of an MCM signal is lost and delay in replication time of an origin, but this correlation is not surprising as an unlicensed origin cannot be an origin and will be replicated passively. What seems most surprising about these data is that the effect is so weak, not that it exists. There is quite a lot of scatter in this plot at 500 uM auxin, with some origins losing a given amount of signal (x) and being only slightly delayed in replication time, and others losing the same amount of signal (x) and being substantially delayed. What underlies this outcome?--Are the ones that are not substantially delayed closer to origins that have not been affected at all by MCM reductions? Why is the correlation so weak? The other regulators of origin activation time have stronger and more precise effects--for example the centromere-control can be precisely eliminated so that only the replication time of the centromere-proximal origins are delayed.

We believe that much of the noise in Figure 4d is due, as the reviewer suggests, to passive replication of origins which lose most of their MCM signal and become inactive but happen to reside next to origins which don’t lost any MCM signal and fire early. And excellent example is ARS 510 (see Figure 4a). ARS510 loses most of its MCM signal and clearly loses its initiation peak in the T_rep plot. However, because it is next to ARS511, which does not lose much MCM signal and which remains a efficient origin, ARS510 is still replicated early. We will include this example in a subsequent revision.

9,Multiple studies in yeast and mammalian cells indicate that MCM subunits are in excess relative to other licensing and S-phase initiation factors, so it is not unexpected that over-expressing MCM did not lead to enhanced levels of licensing. It seems much more plausible that Cdc6 or Cdt1 or both factors are present in limiting amounts for MCM loading, so I did not understand the point of over-producing MCM subunits. If the "weak" origins are the ones that are most dramatically affected by reducing MCM to "limiting" levels, isn't the question whether you can increase licensing at these origins when you over-produce a factor that is likely limiting for licensing, such as Cdt1 or Cdc6 (or both) while leaving MCM at its normal levels. The fact that MCM levels are not limiting for licensing is not surprising and, if anything, argues against these levels having a regulatory role in origin activation timing---which seems to be the opposite of what the authors want to conclude.

Orc1-6, Cdc6 and Cdt1 are all substoichiometric to MCM. However, they all act catalytically to load MCM. So, although they may be kinetically limiting, they do not prevent most or all MCMs being loaded in wild-type cells. The fact that overexpressing MCMs (with or without Cdt1) does not allow for more MCM loading suggests that under normal conditions origins are saturated with MCMs and have little or no capacity to load more MCM, even when given plenty of time to do so. From this result, we conclude that origin capacity is a major determinant of MCM loading in wild-type cells. From our MCM-reduction experiments, we also conclude that, when MCM is limiting, origin competition affects which origins load MCMs faster. However, we agree with the reviewer's first point, that our title gave the incorrect impression that we concluded that origin competition is the primary determinant of MCM loading in wild-type cells. Thus, as suggested, we have changed the title. We have also reworked the Introduction and Discussion to more clearly explain that competition is only a determining factor when MCMs are limited.

In summary, I think the technical aspects of the experiments were quite strong, but I do not think that the experiments answered the question that was posed by the authors.

**Minor points:**

Many places where "This data" should be changed to "These data". Data are plural.

See comments on this point in the response to Reviewer #2.
